# Evaluation criteria for health apps supporting medication adherence in early-stage technology development – a scoping review

**DOI:** 10.3205/000317

**Published:** 2023-04-04

**Authors:** Anja Niemann, Theresa Hüer, Anja Neumann, Jürgen Wasem, Petra Schnell-Inderst, Silke Neusser

**Affiliations:** 1 Institute for Health Care Management and Research, University Duisburg-Essen, Essen, Germany; 2 Institute of Public Health, Medical Decision Making and Health Technology Assessment, Department of Public Health, Health Services Research and Health Technology Assessment, UMIT TIROL – University for Health Sciences and Technology, Hall i.T., Austria

**Keywords:** mobile application, mHealth, health app, drug therapy, systematic review, assessment, criteria

## Abstract

**Introduction:**

Health apps offer an approach to improve the patients’ management of their medication. Although the Digital Healthcare Act (DVG) has created a claim in the statutory health insurance (SHI), the large number of health apps available and their varying quality make it difficult for service providers and especially for medical laypersons to select an adequate high-quality medication app. Manufacturers need guidance for the development of high-quality apps right from the start. Various general evaluation concepts for health apps have been available to date. However, the requirements that should be met by healthcare depend largely on the field of application and the type of apps. This article aims to provide an overview of the international evidence on specific criteria for the evaluation of medication apps.

**Methods:**

Within the framework of a scoping review, a systematic search was conducted in PubMed and EMBASE on January 29, 2020. The search was limited to publications from 2007 onwards as well as to English and German articles. Additionally, a semi-systematic research of reference lists of the previously included articles as well as a structured search of websites of relevant stakeholders were conducted. Inclusion criteria were the following: the publication deals with health apps that can be used on smartphones and focus on supporting medication intake; the publication does not refer to evaluation criteria for a single app exclusively. The included publications were examined in a qualitative content analysis searching for evaluation criteria and categorizing them according to the framework criteria of the DVG and the Digital Health Applications Ordinance (DiGAV).

**Results:**

2,542 articles were identified in the systematic search (999 in PubMed, 1,543 in EMBASE, 560 duplicates). A total of 16 studies met the inclusion criteria. The semi-systematic research and the structured search identified one further study. A catalog of criteria was developed based on the included 17 studies. This catalog covers the general topics “patient orientation” (data protection and security, consumer protection, user friendliness) and “quality/core functions of medication apps” (reminder, self-monitoring, (drug) information, motivation to change behavior, drug/patient safety, robustness) as well as “interoperability/cooperation”. Due to its specific importance for medication apps, the subcategory “motivation for behavioral change” stands out beneath the general topic “quality/core functions of medication apps”. This category aims to evaluate the design of individual functions with regard to their potential to actually change the behavior of app users.

**Discussion:**

The criteria for the evaluation of health apps mentioned in the DiGAV intersected with the criteria identified in the literature research. However, the area of positive health care effects was hardly covered by the included studies. In the development of the criteria catalog, it was not possible to weight the identified criteria. Therefore, the catalog should be understood as a supporting checklist for service providers, manufacturers, and/or users.

**Conclusions:**

A large variety of possible evaluation criteria for medication apps could be shown. Future research should focus on the possibilities of weighting these diverse evaluation criteria, using not only clinical studies but also methods to identify preferences.

## Zusammenfassung

**Einführung:**

Gesundheits-Apps bieten einen möglichen Lösungsansatz zur Verbesserung des Medikamentenmanagements durch Patienten. Zwar wurde mit dem Digitale-Versorgung-Gesetz (DVG) ein Anspruch in der Gesetzlichen Krankenversicherung (GKV) geschaffen, aber die Vielzahl angebotener Gesundheits-Apps mit schwankender Qualität machen es dem Leistungserbringer und vor allem dem medizinischen Laien schwer, eine bedarfsgerechte und qualitativ hochwertige Medikations-App auszuwählen. Auch Hersteller benötigen für die Entwicklung von qualitativ hochwertigen Apps von Anfang an Orientierungshilfen. Bislang liegen diverse allgemeine Bewertungskonzepte für Gesundheits-Apps vor. Welche Anforderungen Gesundheits-Apps erfüllen sollten, hängt jedoch maßgeblich von der Einsatzsituation beziehungsweise dem Typ der App ab. Daher ist die Zielsetzung des vorliegenden Beitrags, einen Überblick über die internationale Evidenzlage zu spezifischen Kriterien zur Bewertung von Medikations-Apps zu schaffen.

**Methoden:**

Im Rahmen eines Scoping Reviews wurde eine systematische Recherche am 29. Januar 2020 in PubMed und EMBASE durchgeführt. Die Suche wurde auf Publikationen ab dem Jahr 2007 und auf englische sowie deutsche Artikel beschränkt. Ergänzt wurde diese durch eine teilsystematische Recherche in den Referenzlisten der zuvor eingeschlossenen Artikel sowie auf Internetseiten von relevanten Stakeholdern. Einschlusskriterien waren: Die Publikation befasst sich mit Gesundheits-Apps, die auf dem Smartphone genutzt werden können und ihren Fokus auf die Unterstützung der Medikamenteneinnahme legen. Zudem durfte die Publikation nicht ausschließlich auf Bewertungskriterien für eine einzelne App bezogen sein. Die identifizierten Publikationen wurden in einer qualitativen Inhaltsanalyse nach Bewertungskriterien untersucht und diese in Anlehnung an die Rahmenkriterien des DVG bzw. der Digitale-Gesundheitsanwendungen-Verordnung (DiGAV) kategorisiert.

**Ergebnisse:**

Die systematische Recherche ergab 2.542 Treffer (999 in PubMed, 1.543 in EMBASE, 560 Dubletten). Insgesamt erfüllten 16 Studien die Einschlusskriterien. Die teilsystematische Recherche identifizierte eine weitere Studie. Der auf Basis der 17 Studien entwickelte Kriterienkatalog umfasst die Oberthemen „Patientenorientierung“ (Datenschutz und -sicherheit, Verbraucherschutz, Nutzerfreundlichkeit) und „Qualität/Kernfunktionen von Medikations-Apps“ (Erinnerung, Selbstüberwachung, (Arzneimittel-)Informationen, Motivation zur Verhaltensänderung, Arzneimittel-/Patientensicherheit, Robustheit) sowie den Bereich „Interoperabilität/Zusammenarbeit“. Aufgrund ihrer spezifischen Bedeutung für Medikations-Apps sticht unter dem Oberthema „Qualität/Kernfunktionen der Medikations-Apps“ die Unterkategorie „Motivation zur Verhaltensänderung“ besonders hervor. Diese zielt auf die Bewertung der Ausgestaltung einzelner Funktionen hinsichtlich ihres Potenzials, das Verhalten der App-Nutzer tatsächlich zu verändern, ab.

**Diskussion:**

Die in der DiGAV genannten Kriterien zur Bewertung von Gesundheits-Apps wiesen eine große Schnittmenge mit den in der Literaturrecherche identifizierten Kriterien auf. Allerdings wurde der Bereich der positiven Versorgungseffekte mit den eingeschlossenen Studien kaum erfasst. Im Rahmen der Entwicklung des Kriterienkatalogs konnte keine Gewichtung der identifizierten Kriterien vorgenommen werden. Daher sollte der Katalog von Leistungserbringern, Herstellern und/oder Nutzern als eine unterstützende Checkliste verstanden werden.

**Fazit:**

Es konnte eine große Vielfalt möglicher Bewertungskriterien für Medikations-Apps gezeigt werden. Künftige Forschung sollte den Fokus auf Möglichkeiten der Gewichtung dieser vielfältigen Bewertungskriterien legen und dafür neben klinischen Studien auch Methoden der Präferenzerhebung einsetzen.

## Background

Insufficient adherence is a widespread problem in drug therapy. The WHO defines adherence as follows: “[Adherence is] the extent to which a person’s behavior – taking medication, following a diet, and/or executing lifestyle changes, corresponds with agreed recommendations from a health care provider” [1]. Studies reveal different, but alarming results. These depend on the indication and patient population considered, as well as on the definition used (exclusively non-adherence or additionally partial adherence). The World Health Organization (WHO) [1] reports that about 50% of medications are not taken as prescribed. Schäfer [2] refers to studies according to which about 20% of all patients do not redeem their prescriptions at all, and half of the remaining 80% of prescriptions are not used at all or not used as prescribed. The consequences of inadequate adherence are complex and affect the health of the patient through medical complications, treatment failure and restrictions on quality of life, but also health care costs and the national economy as a whole. These economic consequences mainly result from increased hospitalization and nursing home admissions, additional costs for therapeutic measures or drugs, and work absenteeism [2]. For example, one in four hospital admissions is reported to be directly or indirectly related to incorrect medication use [2].

The WHO [1] describes forgetting to take the correct dosage as the most common reason for non-adherence. However, numerous other factors also play a role in the correct intake of medications. These include the timing of intake or even foods that are ingested in combination and could trigger interactions. In addition, with the aging population, the proportion of multimorbid individuals taking multiple medications simultaneously is also growing [3]. For example, a U.S. study identifies polypharmacy in 39% for the age group over 65 years. According to the most commonly used definition, polypharmacy is defined as the daily use of five or more medications [4]. However, a significant increase in polypharmacy can also be observed in persons 20 to 39 years old [4]. Adherence in drug therapy can thus become a complex challenge for the individual patient.

One possible approach to improving medication management by patients is offered by health apps to support medication intake (hereinafter medication apps). The smartphone is ubiquitous among many people and can thus be reliably integrated into everyday life. Apps for documenting and reminding patients to take their medications are used increasingly. According to a 2017 survey of 18- to 64-year-olds in Germany conducted by the opinion research company Statista [5], 12% have used a health app within the last twelve months. Specific medication apps offer features such as medication reminders, tracking of doses taken or skipped, an overview of current medication, warnings of drug interactions, medication refill reminders, medication information, and sharing of medication data with third parties [6]. There is evidence that medication apps can improve medication adherence among users. A systematic review included a total of 17 comparative studies examining the effect of medication apps on medication adherence. In twelve of these 17 studies, a significant improvement in adherence was shown for at least one adherence measurement method [7]. With the entry into force of the Digital Health Care Act (DVG) [8] on December 19, 2019, the entitlement of insured persons in the Statutory Health Insurance (SHI) to health apps was anchored in the German Social Code (SGB) V. The health apps must either be prescribed by a contract doctor or psychotherapist or be approved by the health insurer (§§ 33a, 139e SGB V). Due to the high attractiveness of the healthcare market and the reimbursement option recently created by the DVG in the SHI system, numerous providers of health apps can be expected to enter the SHI market. A large number of health apps are already available in the app stores, and this number is expected to grow. The quality of these apps varies [9]. This confusing market already makes it difficult for the medical layperson and will also confront the contract physician with the challenge of selecting a suitable application for his or her needs or those of the app user [9]. The DVG addresses this issue by limiting the claim to health apps that will be included in a directory to be maintained by the Federal Institute for Drugs and Medical Devices (BfArM) after comprehensive review. The Digital Health Applications Ordinance (DiGAV) [10] to the DVG, which entered into force on April 21, 2020, specifies the requirements for digital health apps to be included in the directory according to Section 139e SGB V. A guideline on this procedure prepared by the BfArM [11] includes precise explanations for manufacturers, service providers and users.

Manufacturers need guidance for the development of high-quality apps right from the start [12]. Currently, there is a variety of general evaluation criteria and concepts that manufacturers can use. For example, in October 2019, the Quality Criteria Core Set for Digital Health Apps (DiGA) “AppQ” was published by the Bertelsmann Foundation [13] with the participation of Fraunhofer FOKUS. The development was funded by the German Federal Ministry of Health. This core set consists of 24 criteria organized into the nine topics of *medical quality, positive care effects, data protection, information security, technical quality, consumer protection and fairness, interoperability, user friendliness and motivation, as well as the connection to the healthcare system*. The criteria are applied via a web application to collect self-reported data from providers of DiGA certified as a medical device. The starting point for the development of the “AppQ” core set of quality criteria was the “APPKRI” meta-criteria catalog for the description and evaluation of health apps previously developed by Fraunhofer FOKUS [14], the development of which was also funded by the German Federal Ministry of Health. In addition, there are various international efforts to create transparency in the field of health apps. In some countries, for example, public health institutions offer platforms with a selection of quality-checked health apps. One example is the NHS Digital Apps Library of the National Health Service in the United Kingdom [15], which was published in 2017 and provides users and care providers with a selection of trusted health apps on different topics. A five-step review process assesses requirements in the nine areas of clinical effectiveness (e.g., evidence-based, clarity of purpose and use of the app), regulatory compliance (e.g., declaration as a medical device), clinical safety (e.g., potential adverse effects of the app), privacy and confidentiality (e.g., privacy statement, consent), (IT) security (e.g., data storage, network communication), usability and accessibility, interoperability (e.g., compatibility with other systems), technical stability (e.g., defined plan for product development), and change management (e.g., version control).

However, the requirements that health apps should meet depend largely on the objective of the app (for example, diagnostic support, companionship for various somatic and psychiatric conditions, documentation of health data, support for a healthy lifestyle). For example, medication apps with the goal of promoting adherence focus on changing patient behavior. In order to create a meaningful evaluation basis (in terms of criteria and instruments) for medication apps, systematic as well as supplementary semi-systematic research on the international evidence base has been conducted as part of a scoping review.

## Research questions

The aim of the present study is to examine the extent to which the legal requirements in Germany are consistent with the results on evaluation criteria retrieved from international literature. However, the evaluation criteria to be identified for medication apps should not be considered in isolation, but should always be classified against the background of the interests of users, manufacturers, and healthcare providers. Finally, the extent to which the identified criteria cover relevant areas of the DiGAV will be considered.

## Methodology

This article was based on a two-stage approach. In the first phase, studies were identified that addressed evaluation criteria for medication apps. The second phase involved the development of a criteria catalog and the assignment of the previously identified criteria to it.

In the first phase, studies were sought that focused on the criteria themselves. For this purpose, the scoping review method was used, which aims to provide an overview of the currently available evidence in the literature, without assessment of the quality of the publications or restrictions regarding the type of study. It can also identify gaps in the evidence [16]. First, a systematic literature search was performed in the PubMed and EMBASE databases on January 29, 2020. Search terms on the top-level topics of health apps, medication adherence, and evaluation criteria were linked using the Boolean operator AND. In addition, the search was limited to results from 2007 onwards, the release year of the first iPhone [17], and to English and German articles. Conference transcripts, letters, and notes were excluded. In the next step, duplicates were removed. Subsequent screening of titles and abstracts, as well as subsequent full-text screening was performed independently by two persons. Discrepancies were resolved by discussion. Publications were included if the following criteria were met: the publication addresses health apps that can be used on smartphones and focus on medication adherence support. In addition, the publication had to consider evaluation criteria and tools for medication apps in general and could not be exclusively related to a specific app. Publications were excluded based on the following criteria: abstract or full text were not available; the health apps studied were primarily used for diagnosis, research, or information, aimed at healthcare providers, limited to SMS exchange, or did not represent standalone software. The search terms are detailed in Appendix 1 (Attachment 1) and the inclusion and exclusion criteria are detailed in Appendix 2 (Attachment 1). In addition, a supplementary semi-systematic search was conducted as part of the scoping review. Reference lists of articles included in the systematic search were screened for relevant publications. In addition, websites of stakeholders, associations and public institutions were searched for relevant publications (see Attachment 1, Appendix 3).

The starting point for the second phase was the evaluation criteria for medication apps identified in the first phase, which until then still existed in a loose unsystematic collection. The central added value of this study lies in the development of a catalog of criteria and the assignment of the previously identified individual criteria to this catalog. The starting point for this was the analysis of the DVG [8]. This defines the requirements for digital health applications, which also include health apps, for inclusion in the directory maintained by the BfArM in accordance with § 139e SGB V. These include requirements for safety, functional suitability, quality, data protection and data security, but also the existence of positive effects on care. The details of these requirements are specified in the DiGAV [10]. Two annexes could be taken from this regulation, in which very detailed question catalogs specify the requirements or criteria. These legally defined criteria for the evaluation of health apps were incorporated into the structure of the criteria catalog. The structure derived from the law was adapted and supplemented against the background of the specific medication reference of this study. Only categories for which evaluation criteria could be identified in the literature search were included in the criteria catalog. Categories that were omitted from the criteria catalog with respect to the legally derived structures are explained in more detail in the discussion section. The additions to the structure of the criteria catalog to the categories derived by law resulted from conspicuously frequently mentioned criteria of the included studies that could not be classified in the legal grid. The final criteria catalog was consented by two authors of this paper. In a next step, the included studies were analyzed within the framework of a coding system, and their individual criteria were assigned to the developed criteria catalog. The detailed procedure can be found in Appendix 4 (Attachment 2). This linkage of current legislation with the criteria and categories identified in the scientific literature ascribes relevance to health policy.

## Results

**Figure 1:**
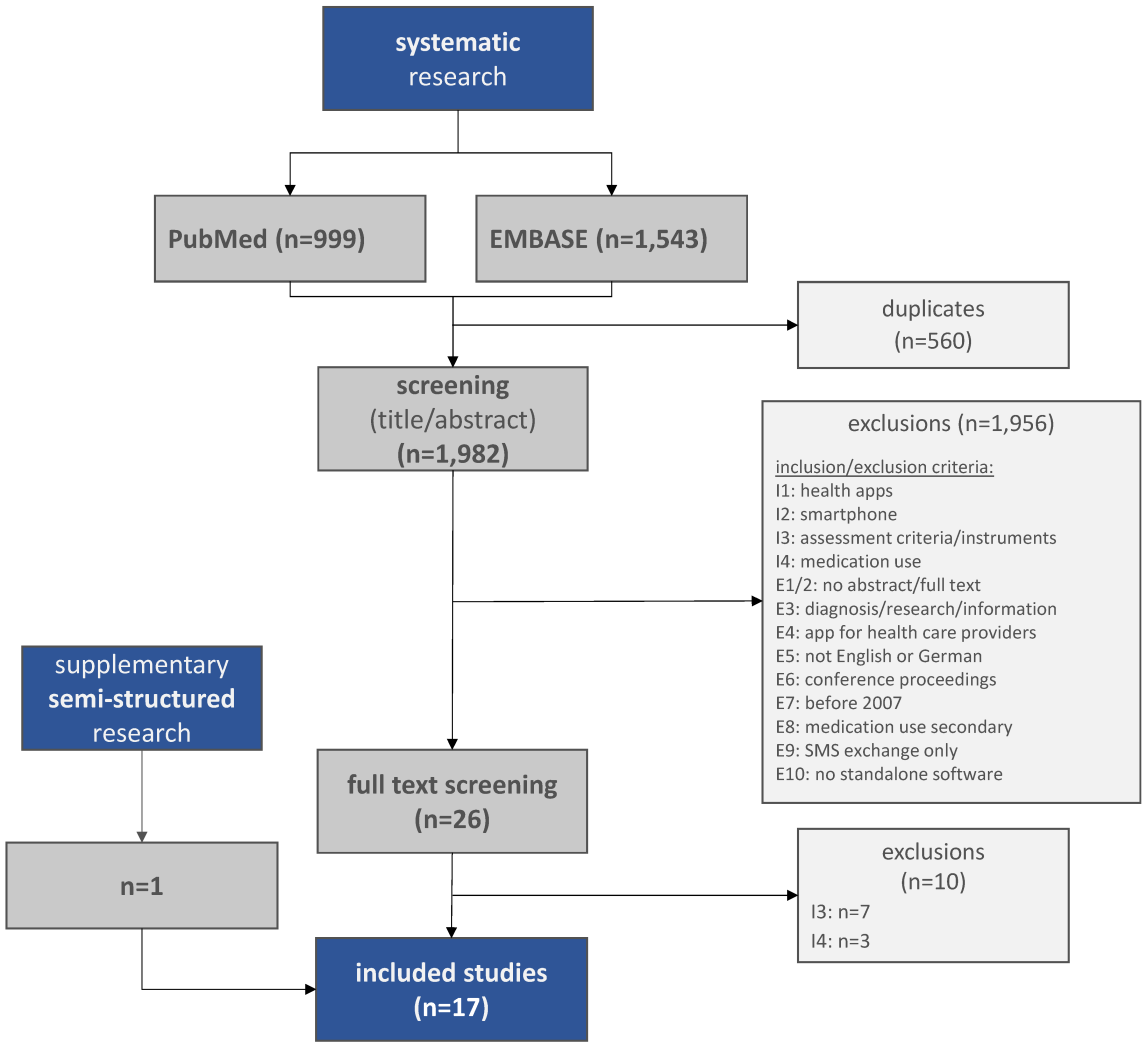
Flowchart of the scoping review

As part of the scoping review, the systematic search identified 999 hits in PubMed and 1,543 hits in EMBASE. After exclusion of 560 duplicates, 1,982 publications entered the title and abstract screening. On this basis, 1,956 publications were excluded because they either did not meet the predefined inclusion criteria and/or met at least one of the exclusion criteria. The inclusion and exclusion criteria can be found in Appendix 2 (Attachment 1) or, in abbreviated form, in the flow chart below (Figure 1 (Fig. 1)). Thus, a total of 26 studies were subjected to full text screening, in which a further ten studies were excluded. One additional study was identified via the supplementary semi-structured search. Thus, a total of 17 studies were included in the content analysis. Since the aim of this paper is to provide an overview of the specific criteria for the evaluation of medication apps that can be taken from the international evidence base, the general evaluation concepts cited in the background which have no direct reference to medication apps are not included in the analysis. However, the general assessment tools used in the included studies (e.g. MARS) are included in the analysis because they are specifically used to assess medication apps in the context of the respective studies. These assessment tools are explained in more detail below.

An overview of the 17 included studies is presented in Table 1 (Tab. 1). In addition to the authors, the year of publication and the country, this overview also shows the methodological approach of the studies and the instruments developed and/or tested for the evaluation of medication apps.

These are predominantly very recent studies (only 2/17 studies were published before 2016) conducted in the USA (n=6), Canada (n=6), Singapore (n=2), China (n=1), Ireland (n=1), and Australia (n=1).

The included studies differ in terms of their methodology with respect to three phases of the development of an assessment tool (see also Table 1 (Tab. 1) for details). In the first phase, criteria for the evaluation of medication apps are identified. A systematic literature review was conducted by two of the included 17 studies [18], [19]. In seven studies the criteria were retrieved from a consensus of the authors and/or semi-systematic searches [6], [20], [21], [22], [23], [24], [25]. Seven other studies adopted previously developed criteria and instruments [26], [27], [28], [29], [30], [31], [32]. One study did not provide information on the development of the criteria list [33]. In the second phase, an assessment instrument is developed based on the identified criteria. In the third phase, the assessment tool developed in each study was applied to evaluate medication apps. Between four [26] and 645 [28] medication apps were included in the different studies (see Table 1 (Tab. 1) for more details). The evaluation of the respective selected apps was partly based only on the app descriptions and screenshots of these and/or by downloading and testing the apps. The evaluation of the medication apps was carried out by the authors of the respective study or medical specialists (n=17) and, in some cases, by the target group itself (n=2) [26], [29].

The last two columns in Table 1 (Tab. 1) describe the evaluation instruments developed for the assessment of apps to support medication adherence. These evaluation instruments were either developed independently or adopted from other studies. Through these instruments, the criteria are measured or operationalized in the included studies.

A total of seven of the 17 included studies developed new instruments for the assessment [6], [18], [20], [33], [21], [19], [25]. In the other studies previously developed instruments were used exclusively (n=7) [26], [27], [28], [29], [30], [31], [32] or in addition to self-developed instruments (n=3) [22], [23], [24]. The simplest assessment instrument is the nominal recording of a criteria. Accordingly, only the presence of certain functions or characteristics is recorded and coded binarily (yes/no or 0=criterion not present/1=criterion present). This operationalization was chosen by five of the ten studies with own contributions in the development of the assessment instrument [6], [33], [23], [24], [25]. The remaining five studies with self-developed instruments operationalized their criteria with varying differentiations. In these studies, individual criteria were weighted. For example, a subsequent weighting in a dichotomous response format was applied (n=3) [20], [21], [22]. In addition, weighting was done with response options to which fixed points are assigned (n=2) [19], [24] and/or Likert scales, i.e., a multilevel response scale with more or less strong agreement or disagreement were used [21]. In addition, a visual rating with one to five stars is used in one study [21]. Often, however, the evaluation instruments consist of a combination of different response formats.

In addition to these newly designed evaluation instruments, established evaluation instruments were used, which were not specifically designed for the evaluation of medication apps. Although these general assessment catalogs are not directly aimed at answering the research question on medication-specific criteria, the MARS instrument, published in 2015, will be briefly discussed below due to the widespread use. It is also available in a version for end users (uMARS). MARS comprises a total of 29 questions in the five areas engagement, functionality, aesthetics, information quality, and subjective quality scale. The questions within the first four areas are measured on a five-point Likert scale (from “inadequate” to “excellent”). The subjective quality assessment is based on predetermined answering options. A mean value is calculated for each area, which is included into the overall evaluation of all mean values [34], [35]. In the studies by Carmody et al. [26] and Morrissey et al. [27], apps are assessed by using a Behavior Change Technique (BCT) catalog [36], [37]. Interventions that aim to change behavior are complex. The challenge is to identify the effective components within the intervention. A BCT describes a component of an intervention that is intended to change behavior, such as reinforcement or feedback. To create BCT catalogs, intervention descriptions from studies that identified techniques associated with effective outcome were used [36]. Medication apps aim to change the user’s behavior to promote adherence. If the concept of the respective app is divided into different components, e.g., reminder function, reward system, or medication list, it is possible to identify the effective components of the apps aiming at behavior change by coding the components according to BCT catalogs. This allows for the differentiation of apps. The presence or absence (0/1) of 26 BCT items is coded in Abraham et al. [37] and of 93 BCT items in Michie et al. [36]. For example, Carmody et al. [26] identify techniques in the apps studied such as detailed planning/definition of behavior (setting alarms, defining medication schedule), identification of environmental cues that remind of desired behavior (setting alarms for a specific time), or recording behavior (recording whether medication dose was taken). In addition to the use of known BCT catalogs, the other included studies (8/17) also recorded elements designed to motivate the user to change behavior [6], [26], [21], [30], [22], [31], [23], [25]. These include, for example, the motivational functions or features “protocol”, “incentives”, and “interaction”. Even though the authors of these studies do not explicitly refer to these evaluation criteria as BCT, they also pursue the objective of identifying components of the tested apps that can change the user’s behavior by evaluating them based on motivational functions and features.

**Figure 2:**
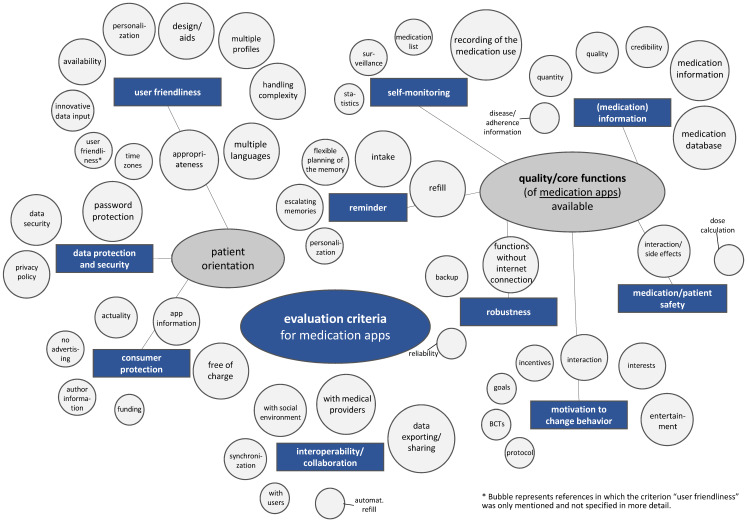
Summary of the evaluation criteria and categorization. Criteria mentioned in only one study are not included in the figure. These include “technical support” (user friendliness), “data transfer to the ePA” (interoperability/collaboration), “recording progress”, “problem solving regarding adherence barriers” (motivation to change behavior), “risk assessment”, “emergency management” (medication/patient safety), and “reminder of physician appointments” (reminder).

The criteria catalog for medication apps developed as part of this study includes the legally defined criteria and combines them with the criteria identified in the literature search. It comprises the overarching topics “patient orientation” and “quality/core functions of medication apps available” as well as the area of “interoperability/collaboration”. The categories “data protection and security”, “consumer protection”, and “user friendliness” are subsumed under the “patient orientation” area. The categories of the topic area “quality/core functions of medication apps available” include “reminder”, “self-monitoring”, “(medication) information”, “motivation for behavior change”, “medication/patient safety”, and “robustness”. In particular, the medication/adherence-specific criteria go beyond the structure of the DiGAV. The criteria identified in the literature review could first be assigned to the categories of the developed criteria catalog and then summarized as a total of 59 evaluation criteria (see also Attachment 2, Appendix 4). This assignment can be seen in Figure 2 (Fig. 2). The size of the bubbles indicates the number of mentions. For example, the criterion “recording of the medication use” with 11/17 mentions is shown significantly larger in Figure 2 (Fig. 2) than the criterion “statistics” with 2/17 mentions.

The wide range of mentions of particular evaluation criteria in the respective studies is striking. Seven individual criteria are each listed in only one of the studies included in this scoping review. They are not shown in Figure 2, but are listed in the legend of Figure 2 (Fig. 2). In contrast, the criterion “recording of the medication use” is mentioned in 11/17 publications. The category “user friendliness” shows a wide variety of evaluation aspects with eleven subcategories, whereas the categories “robustness” and “data protection and security” show a narrow range of criteria with only three criteria each. The included studies place a large focus on the area of core functions of medication apps. Among the wide range of criteria, the presence of functions for “recording of the medication use” (11/17) and “medication database” (9/17) are mentioned particularly frequently. Other frequently mentioned criteria are “data exporting/sharing” (10/17) and “collaboration with medical providers” (8/17) in the “interoperability/collaboration” category, “password protection” (8/17) in the “data protection and security” category, and “appropriateness” (8/17) in the “user friendliness” category. Furthermore, some aspects are mentioned in the studies to support the app user’s motivation to change behavior. Two BCT catalogs have been incorporated, which in turn describe a plethora of behavior change techniques as a stand-alone tool. For example, Michie et al. [36] group 93 BCTs into the following categories: planned consequences, reward and threat, repeat and replace, environment, associations, covert learning, natural consequences, feedback and monitoring, goals and planning, social support, comparing behavior, confidence, comparing outcomes, identity, shaping knowledge, and regulation.

## Discussion

Through the conducted literature search, 59 different criteria for the evaluation of apps for medication adherence support could be identified. In the included studies, 10/17 studies developed assessment tools based on systematic literature review, author consensus, semi-systematic literature search, or in one case without specifying the basis, and/or 10/17 studies used existing generic tools (e.g., MARS or BCT). The 59 identified criteria were assigned to the categories of the criteria catalog developed in this study. This criteria catalog includes the headings “patient orientation” and “quality/core functions of medication apps available” as well as the area “interoperability/collaboration”. The three categories “data protection and security”, “consumer protection” and “user friendliness” are subsumed under the “patient orientation” area. Categories in the topic area “quality/core functions of medication apps available” include six categories of “reminder”, “self-monitoring”, “(medication) information”, “motivation to change behavior”, “medication/patient safety”, and “robustness”. While some of the categories specifically target medication apps, others are also relevant in the evaluation of general health apps.

The category “motivation to change behavior” stands out in the developed criteria catalog due to its specific importance for medication apps and its special design and will be briefly discussed below. This category aims to evaluate the design of individual functions in terms of their potential to actually change the behavior of app users. In two studies [26], [27], detailed question catalogs with evidence-based BCT were used for this purpose. In addition, individual criteria were addressed in eight of the 17 included studies in this area, such as incentives or goal setting. The two included papers using the BCT catalogs assigned the components of the tested apps to specific behavior change techniques (BCT). Via this mapping, it is possible to identify the components of an app that are effective in terms of behavior change. Assuming that motivation for behavior change is one of the main goals of medication apps, a qualitative evaluation of individual components and their specific design can be carried out that way. When designing a medication app, the manufacturer can examine components of the app concerning the criteria of motivation to change behavior and thus focus on particularly effective components and their design.

Since the DiGAV and the BfArM guideline were important starting points for the development of the criteria catalog presented in this study, a brief comparison of the central legal requirements and the included studies will be conducted below. Overall, the appendices of the DiGAV contain comprehensive questionnaires with a higher level of detail than the included studies. These questionnaires serve as a tool for part of the evaluation categories defined by the DiGAV. These include data privacy and security (Appendix 1 of the DiGAV, [10]) as well as interoperability, robustness, consumer protection, user friendliness and accessibility, provider support, medical content quality, and patient safety (Appendix 2 of the DiGAV, [10]). Via yes-no statements as well as the statement “not applicable” the manufacturer confirms the queried criteria. A “not applicable” answer that is not given for selection requires a written explanation of why the criterion has not been fulfilled yet [10]. The category of positive health care effects is explained in the text of the regulation and ranks highly in the DiGAV [10] for inclusion in the Digital Health Applications Directory [10]. According to Section 10 of the DiGAV [10], [11], the manufacturer must demonstrate positive care effects with quantitative comparative studies. These can be clinical or epidemiological studies, but methods from other scientific fields such as health services research, social research, or behavioral research are also permissible, as long as the chosen method fits the subject of the study. Not only prospective, but also retrospective studies (including data from digital patient records, routine data, and registries) are licit. For example, case-control studies, retrospective cohort studies, or even intraindividual comparisons are applicable [10]. In addition to the positive health care effects mentioned in the regulation, the criteria catalog developed in this paper lists individual criteria that correspond to the DiGAV [10] categories of the coordination of treatment processes (e.g., criterion “collaboration with medical providers”), patient safety (e.g., criterion “interaction/side effects”), and health literacy (e.g., “medication information”). For the important categories of prescription adherence and medical benefit (shortening of disease duration, prolongation of survival, improvement of quality of life), however, the criteria catalog does not provide any criterion that directly measures these dimensions. Thus, in the included studies, the assessment of medical effects by criteria plays no or a rather subordinate role. This will be explained in more detail in the following sections. Furthermore, the DiGAV mentions the CE conformity marking as the only aspect for assessing safety and functional suitability. This EU marking is not addressed in the included studies, which were primarily conducted in non-EU countries; however, comparable, general markings are not mentioned either. The category of interoperability and collaboration, which is highlighted in the DiGAV, is also found in the included studies. This involves, for example, resetting an app to its initial state, plausibility checks when entering data, transferring data to the electronic patient record, or sharing data with care providers and the social environment. Overall, it is clear that the structure and content of the criteria catalog developed in this paper based on the included studies show a large overlap with the requirements of the DiGAV. Only the area of positive care effects was hardly covered by the included studies.

For quality assurance purposes, the model developed by Arah et al. [38] in 2006 for the comparative assessment of health care systems in OECD member countries (OECD=Organisation for Economic Co-operation and Development) will be applied to the list of criteria developed in this study. In this model, criteria for assessing the performance of a health care system are grouped into specific dimensions in a condensed list. The comparison shows that the aspects of the OECD model safety of care (esp. via categories “data protection and security”, “consumer protection”, “medication/patient safety”), patient-centeredness (esp. via categories “user friendliness” and “motivation to change behavior”), access to care (esp. category “user friendliness”), and expenditures/costs (esp. criterion “free of charge” in category “consumer protection”) are addressed by the catalog of criteria developed in this study. However, the main segment of effectiveness described in the OECD model cannot be filled with criteria.

The lack of evaluation criteria regarding the effectiveness or positive effects of medication apps on care is due to the fact that the evaluation criteria in the included studies primarily focus on the range of functions and technical features of the apps. This in turn is due to the methodology of the included studies. The evaluation of the selected apps in the third phase of the included studies was partly based only on the app descriptions and screenshots of these and/or by downloading and testing the apps over a very short period of time by the authors and not by users themselves (the latter in only 2/17 studies). Hence, only dimensions that are measurable in the early development phase of the technology and thus can be assessed in the short term can be considered according to the objective of this scoping review. As to the identified criteria, it could not be determined whether and to what extent there are correlations between a particular evaluation criterion and long-term medical benefits. The evaluation of criteria concerning long-term effectiveness would have to be implemented in clinical trials of a comparative nature in which patients or app users are included. Clinical evaluation of care effects could be measured with outcome parameters related to adherence or drug safety/interactions. At the early stages of technology development, systematic reviews of clinical trials on medication apps might help to identify possibly effective app components that might contribute to the improvement of the quality of care. In addition, manufacturers could evaluate medication app components according to the BCT catalogs described earlier in order to gain an impression of the app’s potential in terms of user behavior change.

Furthermore, it should be emphasized that the frequency of criteria mentions does not yet indicate their relevance. It was not possible to weight the identified criteria as part of the development of the criteria catalog. Thus, from this scoping review app developers cannot derive which aspects picking up on the evaluation criteria should be given special emphasis in the design of the app in order to generate the greatest possible medical benefit for the app user. To weight the evaluation criteria identified in this scoping review, further research is essential, particularly in the form of comparative clinical studies that establish and quantify a relationship between the fulfillment of certain criteria and the occurrence of medical effects. This may confirm criteria that are associated with increased patient benefit in the long term. In addition, the relevance of criteria could differ greatly with respect to the perspective taken. Therefore, preference surveys, such as choice-based conjoint analyses, could be a useful addition as a basis for weighting criteria. In these, alternatives could be evaluated as a bundle of their characteristics (i.e., specific evaluation criteria) from different perspectives (service provider, user, payer) in an intuitive decision-making process. For example, from the perspective of the treating physician, criteria that aim at an efficient (data) exchange with the patient and evaluate the effectiveness of the medication app could be important. Users, on the other hand, might consider criteria that evaluate the presentation of medical content and the handling of the app to be more important. A mapping of preferences to the criteria identified in this study with regard to different target groups could improve the use as a targeted basis for decision-making.

## Limitations

In the included studies, the mentioned criteria are presented in different forms, for example as a sentence or only as a short bullet point, and different formulations for criteria are chosen. The summary of the criteria of the included studies into unified evaluation criteria can be differentiated to varying degrees. With this in mind, a balance between detailed differentiation and clear summary was sought in this study and developed by consensus as understood by the authors of this paper. This also applies to the chosen structure of the criteria catalog, which is based on the results found in the studies in addition to the structure of the DiGAV. Moreover, in the context of a scoping review, the evidence from the literature is reported regardless of the quality of the included studies, so that all criteria found were equally mentioned and summed up. Furthermore, the selected inclusion and exclusion criteria of the literature search were used to identify studies that addressed the design of evaluation criteria/instruments for medication apps in order to apply them to the evaluation of a set of different apps. Studies that only addressed the evaluation of a specific app were excluded. It is possible that evaluation criteria could have been indirectly derived from these publications as well.

## Answering the research questions

The criteria for evaluating health apps identified in the DiGAV had a high degree of intersection with the criteria identified in the literature review. However, the area of positive care effects was poorly captured with the included studies. Quality assurance of the criteria catalog via the OECD model according to Arah et al. [38] revealed that the aspects of safety of care, patient-centeredness, access to care, and expenditures/costs were covered via the developed criteria catalog. The main segment of effectiveness described in the OECD model, however, could also not be fulfilled due to the short-term dimension or the rather functional orientation of the identified evaluation criteria. In addition, the frequency of mentions of the criteria alone does not indicate the relevance of a criterion. For these reasons, it was not possible to weight the identified criteria during the development of the criteria catalog. Therefore, the criteria catalog developed should not be understood by service providers, manufacturers and/or users as a comprehensive evaluation tool with a clear result, but rather as a supporting checklist. It is thus only a first step in the development of decision support tools.

Important potentials are associated with the criteria catalog developed in this article. In order to be included in the list of reimbursable digital health applications maintained by the BfArM in accordance with § 139e SGB V, health apps must fulfill the required conditions of the DiGAV [10], which are explained by the BfArM by means of examples in the DiGAV guide. In addition to the manufacturer’s specifications, the list also includes information on the features and performance of the digital health apps listed [11]. If the healthcare provider or a patient himself [11] is faced with the challenge of selecting the most suitable registered application, an evaluation that goes beyond the register and is more specific to the area of medication management plays an important role. The evaluation criteria identified in the present study can serve as a basis for a qualitative evaluation of medication apps and thus provide a supplementary decision-making aid for healthcare providers and also for potential app users for the needs-based selection of medication apps that have been included in the register of reimbursable digital health apps.

## Conclusion

In view of the ever scarcer resources in the health care system, the efficient use of drugs is essential. Schäfer [2] states that the most expensive drug is the one that the patient does not take. Therefore, digital approaches promoting adherence should be focused on. However, medical care providers, manufacturers and users need more specific guidance than the general concepts available to date. In the present scoping review, an overview of the international evidence base on criteria for the evaluation of medication apps could be created and presented in a structured way in a criteria catalog. In addition to the studies included in the literature search, the legal requirements in Germany (DVG, DiGAV) in particular served as the basis for the development of this criteria catalog. Future research should focus on ways to weight these criteria, using preference survey methods in addition to clinical studies. Furthermore, dimensions of effectiveness or positive effects of care should be added to the criteria catalog.

## Abbreviations

- BCT: Behavior Change Technique
- BfArM: Bundesinstitut für Arzneimittel und Medizinprodukte (Federal Institute for Drugs and Medical Devices)
- DiGA: Digitale Gesundheitsanwendung (digital health application)
- DiGAV: Digitale-Gesundheitsanwendungen-Verordnung (Digital Health Applications Ordinance)
- DVG: Digitale-Versorgung-Gesetz (Digital Healthcare Act)
- ePA: Elektronische Patientenakte (electronic patient file)
- SHI: Statutory health insurance
- MARS: Mobile App Rating Scale
- Medikations-App: Gesundheitsapps zur Unterstützung der Medikamenteneinnahme (health app to support intake of medications)
- NHS: National Health Service (Great Britain)
- OECD: Organisation für wirtschaftliche Zusammenarbeit und Entwicklung (Organisation for Economic Co-operation and Development)
- SGB: Sozialgesetzbuch (social security code)
- uMARS: User Version of the Mobile Application Rating Scale
- WHO: World Health Organization

## Notes

### Funding

The project was supported by the German Institute of Medical Documentation (DIMDI).

### Competing interests

The authors declare that they have no competing interests.

## Supplementary Material

Appendix 1–3

Appendix 4

## Figures and Tables

**Table 1:**
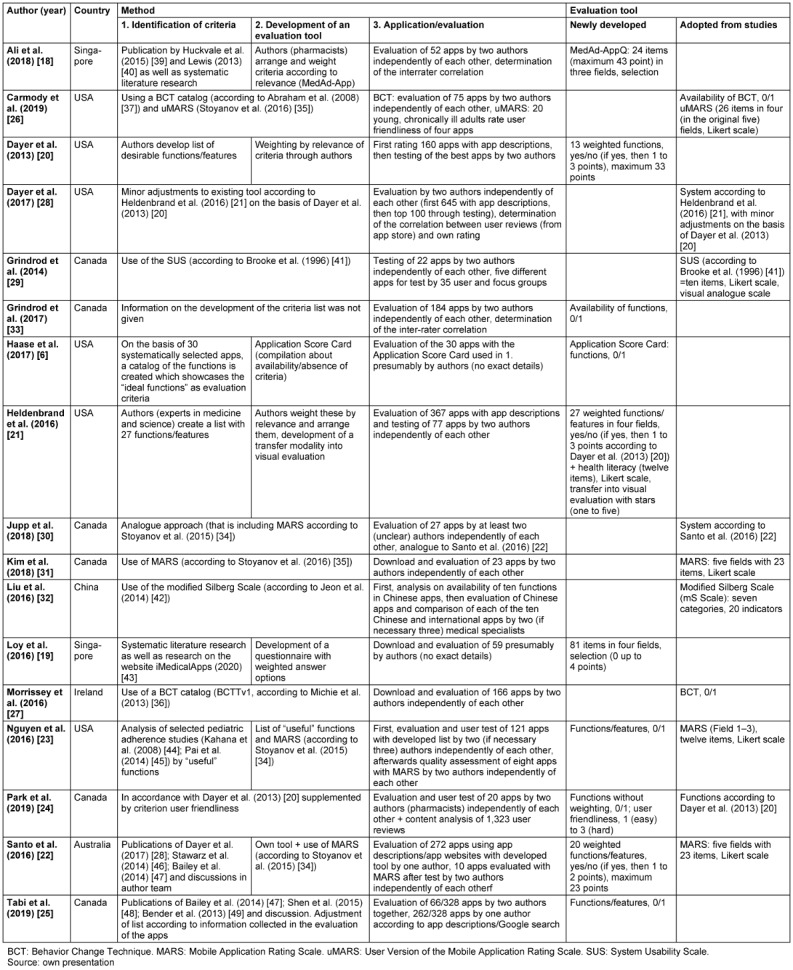
Overview of the studies included in this scoping review
