# Supplementary material for: Evaluation criteria for health apps supporting medication adherence in early-stage technology development – a scoping review
Source: Ger Med Sci. 2023 Apr 4;21:Doc03. doi: 10.3205/000317 (PMC10227643; doi:10.3205/000317)
Supplement: Appendix 1–3 [file GMS-21-03-s-001.pdf]

Attachment 1

Appendix 1: Presentation of the search strategy in EMBASE and PubMed

EMBASE (01/29/2020)

(‘mobile application’/exp OR ‘mobile application’ OR ‘health app*’ OR ‘mobile app*’ OR ‘smartphone app*’ OR ‘app store’ OR ‘play store’ OR ‘google play’ OR ‘portable software app*’ OR ‘medical app’ OR ‘medical apps’ OR ‘medical application’ OR ‘medical applications’ OR ‘mHealth’)
AND
(‘medication’/exp OR medication OR ‘drug therapy’/exp OR ‘drug therapy’ OR ‘drug therap*’ OR ‘pharmacological treatment*’ OR ‘medicament therapy’ OR ‘medicament treatment’ OR ‘pharmaceutical therapy’ OR ‘pharmaceutical treatment’ OR ‘pharmaco-therapy’ OR ‘pharmaco-treatment’ OR ‘pharmacological therapy’ OR ‘pharmacotherapy’ OR ‘pharmacotreatment’ OR ‘therapeutic uses’ OR ‘therapy, drug’ OR ‘therapy, pharmacological’ OR ‘treatment, drug’ OR ‘treatment, pharmacological’)
AND
(assess* OR evaluat* OR criteria* OR rating* OR scale* OR ‘quality’/exp OR quality)
AND
([2007-2020]/py AND ([english]/lim OR [german]/lim) NOT [conference paper]/lim NOT [conference review]/lim NOT [conference abstract]/lim NOT [letter]/lim NOT [note]/lim)

PubMed (01/29/2020)

(“mobile applications”[MeSH Terms] OR “mobile application” OR “mobile applications” OR “mobile app” OR “mobile apps” OR “Health application” OR “Health applications” OR “Health app” OR “Health apps” OR “smartphone app” OR “smartphone apps” OR “smartphone application” OR “smartphone applications” OR “app store” OR “play store” OR “google play” OR “portable software app” OR “portable software apps” OR “portable software application” OR “portable software applications” OR “medical app” OR “medical apps” OR “medical application” OR “medical applications” OR “mHealth”)
AND
(Medication[MeSH Terms] OR medication OR “drug therapy”[MeSH Terms] OR “drug therap*” OR “drug therapy” OR “pharmacological treatment” OR “pharmacological treatment*” OR “medicament therapy” OR “medicament treatment” OR “pharmaceutical therapy” OR “pharmaceutical treatment” OR “pharmaco-therapy” OR “pharmaco-treatment” OR “pharmacological therapy” OR “pharmacotherapy” OR “pharmacotreatment” OR “therapeutic uses”)
AND
(assess* OR assessments OR assessment OR evaluat* OR evaluation OR evaluations OR criteria OR criterias OR criteria* OR rating* OR rating OR ratings OR scale OR scales OR scale* OR quality)
AND
(“2007/01/01”[PDat] : “3000/12/31”[PDat]) NOT (Clinical Conference[ptyp] OR Congress[ptyp] OR Letter[ptyp]) AND (English[lang] OR German[lang]) AND (full text[sb])

Appendix 2: Presentation of in- and exclusion criteria

Inclusion criteria

- **I1** Topic area health apps.
- **I2** Health app can be used on smartphone.
- **I3** Publication deals with general evaluation criteria and tools.
- **I4** Health app focuses on support of medication intake/medication in general.

Exclusion criteria

- **E1** No abstract available.
- **E2** No full text available.
- **E3** Health app used primarily for diagnosis, research/information.
- **E4** Health app is directed towards service provider.
- **E5** Language of the publication is not English or German.
- **E6** Publication is a conference paper, review, abstract, a letter, a note.
- **E7** Publication before 2007.
- **E8** Support for taking medications is only a subordinate part of the health app.
- **E9** Mobile use is limited to SMS exchange.
- **E10** Health app is no standalone software, but one that e.g. is a control software for another medical device.

Attachment 1 to: *Niemann A, Hüer T, Neumann A, Wasem J, Schnell-Inderst P, Neusser S. Evaluation criteria for health apps supporting medication adherence in early-stage technology development – a scoping review. GMS Ger Med Sci. 2023;21:Doc03. DOI: 10.3205/000317, URN: urn:nbn:de:0183-0003171*

Appendix 3: List of the sources used in the semi-systematic research

I. Reference list of included studies

- Ali EE, Teo AKS, Goh SXL, Chew L, Yap KY. MedAd-AppQ: A quality assessment tool for medication adherence apps on iOS and android platforms. Res Social Adm Pharm. 2018 Dec;14(12):1125-33. DOI: 10.1016/j.sapharm.2018.01.006
- Carmody JK, Denson LA, Hommel KA. Content and Usability Evaluation of Medication Adherence Mobile Applications for Use in Pediatrics. J Pediatr Psychol. 2019 Apr;44(3):333-42. DOI: 10.1093/jpepsy/jsy086
- Dayer L, Heldenbrand S, Anderson P, Gubbins PO, Martin BC. Smartphone medication adherence apps: potential benefits to patients and providers. J Am Pharm Assoc (2003). 2013;53(2):172-81. DOI: 10.1331/JAPhA.2013.12202
- Dayer LE, Shilling R, Van Valkenburg M, Martin BC, Gubbins PO, Hadden K, Heldenbrand S. Assessing the Medication Adherence App Marketplace From the Health Professional and Consumer Vantage Points. JMIR Mhealth Uhealth. 2017 Apr;5(4):e45. DOI: 10.2196/mhealth.6582
- Grindrod KA, Li M, Gates A. Evaluating user perceptions of mobile medication management applications with older adults: a usability study. JMIR Mhealth Uhealth. 2014 Mar;2(1):e11. DOI: 10.2196/mhealth.3048
- Grindrod K, Boersema J, Waked K, Smith V, Yang J, Gebotys C. Locking it down: The privacy and security of mobile medication apps. Can Pharm J (Ott). 2017;150(1):60-6. DOI: 10.1177/1715163516680226
- Haase J, Farris KB, Dorsch MP. Mobile Applications to Improve Medication Adherence. Telemed J E Health. 2017 Feb;23(2):75-9. DOI: 10.1089/tmj.2015.0227
- Heldenbrand S, Martin BC, Gubbins PO, Hadden K, Renna C, Shilling R, Dayer L. Assessment of medication adherence app features, functionality, and health literacy level and the creation of a searchable Web-based adherence app resource for health care professionals and patients. J Am Pharm Assoc (2003). 2016;56(3):293-302. DOI: 10.1016/j.japh.2015.12.014
- Jupp JCY, Sultani H, Cooper CA, Peterson KA, Truong TH. Evaluation of mobile phone applications to support medication adherence and symptom management in oncology patients. Pediatr Blood Cancer. 2018 Nov;65(11):e27278. DOI: 10.1002/pbc.27278
- Kim BY, Sharafoddini A, Tran N, Wen EY, Lee J. Consumer Mobile Apps for Potential Drug-Drug Interaction Check: Systematic Review and Content Analysis Using the Mobile App Rating Scale (MARS). JMIR Mhealth Uhealth. 2018 Mar;6(3):e74. DOI: 10.2196/mhealth.8613
- Liu Y, Chang P, Li N, Lamb KV, Wang J, Huang X, Wang Y, Wu Y. Quality of Chinese CVD Medication Apps. Stud Health Technol Inform. 2016;225:1036-7.
- Loy JS, Ali EE, Yap KY. Quality Assessment of Medical Apps that Target Medication-Related Problems. J Manag Care Spec Pharm. 2016 Oct;22(10):1124-40. DOI: 10.18553/jmcp.2016.22.10.1124
- Morrissey EC, Corbett TK, Walsh JC, Molloy GJ. Behavior Change Techniques in Apps for Medication Adherence: A Content Analysis. Am J Prev Med. 2016 May;50(5):e143-e146. DOI: 10.1016/j.amepre.2015.09.034
- Nguyen E, Bugno L, Kandah C, Plevinsky J, Poulopoulos N, Wojtowicz A, Schneider KL, Greenley RN. Is There a Good App for That? Evaluating m-Health Apps for Strategies That Promote Pediatric Medication Adherence. Telemed J E Health. 2016 Nov;22(11):929-37. DOI: 10.1089/tmj.2015.0211
- Park JYE, Li J, Howren A, Tsao NW, De Vera M. Mobile Phone Apps Targeting Medication Adherence: Quality Assessment and Content Analysis of User Reviews. JMIR Mhealth Uhealth. 2019 Jan;7(1):e11919. DOI: 10.2196/11919

- Santo K, Richtering SS, Chalmers J, Thiagalingam A, Chow CK, Redfern J. Mobile Phone Apps to Improve Medication Adherence: A Systematic Stepwise Process to Identify High-Quality Apps. JMIR Mhealth Uhealth. 2016 Dec;4(4):e132. DOI: 10.2196/mhealth.6742
- Tabi K, Randhawa AS, Choi F, Mithani Z, Albers F, Schnieder M, Nikoo M, Vigo D, Jang K, Demlova R, Krausz M. Mobile Apps for Medication Management: Review and Analysis. JMIR Mhealth Uhealth. 2019 Sep;7(9):e13608. DOI: 10.2196/13608

II. Websites of stakeholders, associations and public institutions

- Public institutions (ministries and downstream authorities/public corporations):
    - Bundesbeauftragte für den Datenschutz und die Informationsfreiheit (Federal Commissioner for Data Protection and Freedom of Information). Available from: https://www.bsi.bund.de/DE/Home/home_node.html
    - Bundesinstitut für Arzneimittel und Medizinprodukte (The Federal Institute for Drug and Medical Devices). Available from: https://www.bfarm.de/DE/Home/_node.html
    - Bundesministerium für Gesundheit (The Federal Ministry of Health). Available from: https://www.bundesgesundheitsministerium.de/
    - NHS England. Available from: https://www.nhs.uk/
    - Techniker Krankenkasse (TK health insurance), TUMAINI-Institut für Präventionsmanagement GmbH. Check die App. Available from: https://www.tk-checkdieapp.de/start.php
- Associations/foundations/institutes/companies:
    - Aktionsbündnis Patientensicherheit (Patient Safety Action Alliance), Patientensicherheit Schweiz (Patient Safety Switzerland), Plattform Patientensicherheit (Platform Patient Safety). Digitalisierung und Patientensicherheit: Checkliste für die Nutzung von Gesundheits-Apps. Available from: https://www.aps-ev.de/wp-content/uploads/2018/05/2020_APS-Checkliste-GesundheitsApps.pdf
    - AOK Bundesverband (AOK Federal Association). Nutzen und Grenzen von Gesundheits-Apps. Available from: https://www.aok-bv.de/presse/medienservice/ratgeber/index_20688.html
    - Bertelsmann Stiftung (Bertelsmann Foundation). AppQ: Gütekriterien-Kernset für mehr Qualitätstransparenz bei digitalen Gesundheitsanwendungen. Available from: https://www.bertelsmann-stiftung.de/de/publikationen/publikation/did/appq
    - DiaDigital. Available from: https://www.diadigital.de/
    - Fraunhofer-Institut für Offene Kommunikationssysteme FOKUS (Frauenhofer Institute for Open Communication Systems). Available from: https://www.fokus.fraunhofer.de/
    - Verband der Ersatzkassen (Association of the Substitute Health Insurances). Ersatzkassen fordern Qualitätsprüfung onlinebasierter Therapieangebote. Available from: https://www.vdek.com/presse/pressemitteilungen/2018/qualitaetspruefung-onlinebasierter-therapieangebote.html
    - Verbraucherzentrale NRW e.V. (Consumer Advice Center North Rhine-Westphalia). Gesundheits-Apps: medizinische Anwendungen auf Rezept. Available from: https://www.verbraucherzentrale.de/wissen/gesundheit-pflege/aerzte-und-kliniken/gesundheitsapps-ab-2020-kommen-medizinische-anwendungen-auf-rezept-41241
    - ZTG Zentrum für Telematik und Telemedizin (Center for Telematics and Telemedicine). Available from: https://www.ztg-nrw.de/
