# Supplementary material for: Evaluation criteria for health apps supporting medication adherence in early-stage technology development – a scoping review
Source: Ger Med Sci. 2023 Apr 4;21:Doc03. doi: 10.3205/000317 (PMC10227643; doi:10.3205/000317)
Supplement: Appendix 4 [file GMS-21-03-s-002.pdf]

Attachment 2

Appendix 4: Overview of the criteria identified in the included studies

study			patient orientation			interoperability/ collaboration	quality/core functions (of medication apps) available					
			**privacy and security**	**consumer protection**	**user friendliness**		**reminder**	**self-monitoring**	**(medication) information**	**motivation to change behavior**	**medication/ patient safety**	**robustness**
1	**Ali et al.**	2018	- data protection regulations/ information on the use of user data (3)	- information about the financial support of the app (6)<br>- information about the developer (name, contact information, references, conflicts of interests) (2)<br>- description of purpose and target group (5)	- convenient data entry (auto-completion, text recognition, barcode) (2)	- share medication lists and intake with third parties (care providers, family, caregivers) (1, 3, 4)<br>- reminders to third parties (4)<br>- alarm that informs third when dose missed (4)	- adjust to complex medication schedules/ plan dose reductions (3)<br>- refill reminder (2)<br>- doctor appointment reminder (6)<br>- snooze (4)	- adherence monitoring: recording of medication intake (1)<br>- medication list (2)	- functions for better medication identification (2)<br>- medication information (1)<br>- disease-related information (3)		- medication side effects/undesirable medication effects (1)	- reminders without internet connection (1)
2	**Carmody et al.**	2019			- personalization (9)<br>- appropriateness (4)<br>- complexity, navigation (1)<br>- design, layout, graphics, appealing look, visual information (3)				- quality of the information (5)<br>- quantity of the information (6)<br>- credibility of the source (4)	- entertainment (1)<br>- interests (2)<br>- interactivity (3)<br>- BCT catalog (inter alia linking behavior and health, consequences) (5)		
3	**Dayer et al.**	2013	- privacy policy (3)	- free (3)	- multilingual (5)<br>- use of multiple profiles (6)<br>- online access to data (2)<br>- multiple platforms (8)	- data synchronization/ export/printing (1)<br>- data input possible by provider (3)		- monitoring of medication intake (1)	- medication database (2)			- reminders without internet connection (1)<br>- data storage in cloud (2)

4	**Dayer et al.**	2017	- passwords (1)<br>- privacy policy (3)	- free (3)	- multilingual (5)<br>- considering different health competencies (4)<br>- use of multiple profiles (6)<br>- multiple platforms (8)	- data exporting/sharing (1)<br>- refill by special pharmacy (7)<br>- connect with other users (5)	- customizable reminder (3)<br>- refill reminder (2)<br>- snooze (4)<br>- medication reminder (1)	- monitoring of medication intake (1)	- medication database (2)		- interaction with food and drugs (1)	- reminders without internet connection (1)<br>- data storage in cloud (2)
5	**Grindrod et al.**	2014			- complexity/ integration of functions/ consistency (1)<br>- technical support (10)							
6	**Grindrod et al.**	2017	- password protection (1)<br>- privacy policy (3)<br>- encryption/ private alarms (2)		- storage of personal health information (9)	- sharing data with third parties (3/4)						
7	**Haase et al.**	2017	- safety (2)			- synchronization (6)<br>- sharing (1)<br>- integration with medical record (2)	- refill reminder (2)<br>- reminders (1)	- medication list (2)<br>- logging (1)	- education (1)	- gamification (1)	- medication interactions (1)	
8	**Helden-brand et al.**	2016	- privacy policy (3)<br>- password protection (1)	- free (3)<br>- no advertising (4)<br>- recent revision (1)	- simple language, precise information, meaningful headings (4)<br>- easy start-up (1)<br>- clearly legible representations, medication photos by the user (3)<br>- multiple languages (5)<br>- use of multiple profiles (6)<br>- online data entry (2)<br>- multiple platforms (8)	- data exporting/ sharing (1)<br>- service provider can provide data/information (3)<br>- submit refill requests (7)<br>- networking with other users (5)	- medication specific reminders (1)<br>- snooze/escalating reminders (4)<br>- customizable reminders (3)<br>- refill alerts (2)	- recording or medication use (1)<br>- detailed anamnesis (3)	- medication database (2)<br>- complex medication Instructions (1)	- incentives (4)	- identification of potential interactions (1)	- reminders without internet connection (1)<br>- data storage in cloud (2)

9	**Jupp et al.**	2018	- data security (2)<br>- password protection (1)	- free (3)<br>no advertising (4)<br>- recent revision (1)<br>- accuracy of description/ objectives (5)	- available in both app stores (8)<br>- use of multiple profiles (6)<br>- multilingual (5)<br>- time zone support (7)<br>- personalization (9)<br>- appropriateness (4)<br>- handling/ navigation (1)<br>- design, layout, graphics, attractive optics, visual info	- data export/printing (1)<br>- notifications to other people (3/4)	- flexible scheduling (3)<br>- snooze (4)<br>- refill reminder (2)<br>- customizable alert tones (5)	- storage of medication intake (1)<br>- statistics/ tables on adherence (4)	- medication database (2)<br>- quality of information (5)<br>- quantity of information (6)<br>- credibility of source (4)<br>- evidence base (4)	- reward for Adherence (4)<br>- entertainment (1)<br>- interests (2)<br>- Interactivity (3)		- reminders without internet connection (1)
10	**Kim et al.**	2018		- accuracy of description/ objectives (5)	- personalization (9)<br>- appropriateness (4)<br>- handling/ navigation (1)<br>- design, layout, graphics, appealing looks, visual information (3)				- quality of the information (5)<br>- quantity of the information (6)<br>- credibility of the source (4)<br>- evidence base (4)	- entertainment (1)<br>- interests (2)<br>- interactivity (3)		
11	**Liu et al.**	2016	- privacy (1)	- authorship (2)<br>- attribution (5)<br>- disclosure (6)<br>- timeliness (1)	- user friendliness (11)<br>- appropriateness (4)	- device linkage (6)<br>- pharmacist advice (3)<br>- social support (4)	- medication reminder (1)	- documentation of the medication (1)	- search for medications (2)<br>- medication information (1)		- risk assessment (2)	
12	**Loy et al.**	2016	- privacy (1)		- user friendliness (11)			- monitoring (1)<br>- medical record (3)	- medical information (1)		- identification of potential interactions (1)<br>- dose calculation (3)	- reliability (3)
13	**Morrissey et al.**	2016								- BCT catalog (including planned consequences, reward and threat) (5)		

14	**Nguyen et al.**	2016			- personalization (9)<br>- appropriateness (4)<br>- handling/ navigation (1)<br>- design, layout, graphics, appealing looks (3)	- sharing revenue records (1)	- timed medication reminders (1)	- save medication names/dosage (2)<br>- record medication intake and reasons for skipping (1)	- educational information about medication (1)<br>- side effects of medication (1)<br>- disease-specific information (3)<br>- benefit of adherence (3)<br>- risk of non-compliance (3)	- entertainment (1)<br>- interests (2)<br>- interactivity (3)<br>- tracking progress (7)<br>- medication goal setting (6)<br>- encouraging intake logging (8)<br>- encouraging problem solving of adherence barriers (9)		
15	**Park et al.**	2019	- backup/cloud (2)	- free (3)	- user friendliness (11)<br>- use of multiple profiles (6)<br>- multilingual (5)	- data export/printing (1)	- ingestion alarm (1)<br>- refill reminder/ remaining stock indicator (2)	- recording of medication intake/storage of medication information (1)	- medication database (2)<br>- complex medication instructions or notes (1)			- reminders without internet connection (1)<br>- access via other end device/cloud (2)
16	**Santo et al.**	2016	- data security (2)<br>- password protection (1)	- free (3)<br>- no advertisement (4)<br>- recent revision (1)<br>- accuracy of description/ goals (5)	- available in both app stores (8)<br>- use of multiple profiles (6)<br>- multilingual (5)<br>- time zone support (7)<br>- personalization (9)<br>- appropriateness (4)<br>- handling/ navigation (1)<br>- design, layout, graphics, attractive optics, visual info	- data exporting/sharing (1)<br>- notifications for third parties (3/4)	- flexible scheduling (3)<br>- snooze (4)<br>- refill reminder (2)<br>- customizable alert tones (5)	- storage of medication intake (1)<br>- statistics/ tables on adherence (4)	- medication database (2)<br>- quality of information (5)<br>- quantity of information (6)<br>- credibility of source (4)<br>- evidence base (4)	- reward for adherence (4)<br>- entertainment (1)<br>- interests (2)<br>- interactivity (3)		- reminders without internet connection (1)
17	**Tabi et al.**	2019	- data privacy (1)<br>- data security (2)	- affiliation (2)<br>- cost (3)	- multiple profiles (6)<br>- multiple languages (5)<br>- innovative data entry (2)<br>- support with time zones (7)<br>- user interface (3)	- sharing data with third parties (1/4)<br>- communication with med. care providers (3)<br>- synchronization with other devices (6)	- medication reminders (1)<br>- personalized voice reminders (5)	- monitoring (symptoms, side effects, health data, vital information)/ correlations based on entered data (3)	- recognizing pills (2)<br>- information about medication (1)	- goal setting (6)<br>- journal (8)	- safety plan for acute emergency situation, emergency button (4)<br>- warning when exceeding the safe dose (3)<br>- interactions (1)	- reliability (3)

**summary**<br>(criteria per category,<br>(responses*/<br>all studies))	1) password protection (8/17)<br>2) data security (6/17)<br>3) privacy policy (5/17)	1) actuality (4/17)<br>2) author information (3/17)<br>3) free of charge (7/17)<br>4) ad-free (3/17)<br>5) app information (5/17)<br>6) funding (2/17)	1) handling complexity (7/17)<br>2) innovative data entry (4/17)<br>3) design/aids (7/17)<br>4) appropriate-ness (8/17)<br>5) multiple languages (7/17)<br>6) multiple profiles (7/17)<br>7) time zone support (3/17)<br>8) availability (Android, iOS) (5/17)<br>9) personaliza-tion (6/17)<br>10) technical support (1/17)<br>11) general user-friendliness (3/17)	1) data export/ sharing (10/17)<br>2) data transfer in ePA (1/17)<br>3) cooperation with med. care providers (8/17)<br>4) collaboration with social environment (6/17)<br>5) exchange with users (2/17)<br>6) synchronization (3/17)<br>7) automatic refill request (2/17)	1) reminder to take (7/17)<br>2) reminder to refill (7/17)<br>3) flexible scheduling of reminder (5/17)<br>4) escalating reminders/ snooze (5/17)<br>5) personaliza-tion (3/17)<br>6) reminder for doctor appointments (1/17)	1) recording of medication intake (11/17)<br>2) list of medications/ incl. dosage (3/17)<br>3) monitoring of health status (3/17)<br>4) statistics on adherence (2/17)	1) medication Information (8/17)<br>2) medication database/ medication identification (9/17)<br>3) disease Information/ adherence (2/17)<br>4) credibility of source (4/17)<br>5) quality of Information (4/17)<br>6) quantity of Information (4/17)	1) entertainment/ gamification (6/17)<br>2) interests (5/17)<br>3) interactivity (5/17)<br>4) incentives (3/17)<br>5) application of BCTs (2/17)<br>6) goal setting (2/ 17)<br>7) recording of progress (1/17)<br>8) encouraging logging (2/17)<br>9) problem solving regarding adherence barrier (1/17)	1) interactions/ side effects (6/17)<br>2) risk assessment (1/17)<br>3) dose calculation including warning (2/17)<br>4) emergency management (1/17)	1) reminders without internet connection (7/17)<br>2) data storage in cloud (4/17)<br>3) reliability (2/17)

* If several aspects in a study can be assigned to one criterion, the fulfillment of the criterion is only counted once, so that a maximum value of (17/17) can be achieved for each criterion.
